# Diagnostic performance of LI-RADS version 2018 in differentiating hepatocellular carcinoma from other hepatic malignancies in patients with hepatitis B virus infection

**DOI:** 10.17305/bjbms.2019.4576

**Published:** 2020-08

**Authors:** Shuo Shao, Yingying Liang, Sichi Kuang, Jingbiao Chen, Qungang Shan, Hao Yang, Yao Zhang, Bin Wang, Kathryn J. Fowler, Jin Wang, Claude B. Sirlin

**Affiliations:** 1 Department of Radiology, The Third Affiliated Hospital, Sun Yat-Sen University (SYSU), Guangzhou, China; 2 Department of Radiology, Jining No.1 People’s Hospital, Jining, China; 3 Affiliated Jining No. 1 People’s Hospital of Jining Medical University, Jining Medical University, Jining, China; 4 Department of Radiology, Guangzhou First People’s Hospital, Guangzhou Medical University, Guangzhou, China; 5 The Second Affiliated Hospital, South China University of Technology, Guangzhou, China; 6 Medical Imaging Research Institute, Binzhou Medical University, Yantai, China; 7 Liver Imaging Group, Department of Radiology, University of California at San Diego, La Jolla, California, USA

**Keywords:** Hepatocellular carcinoma, HCC, intrahepatic cholangiocarcinoma, ICCA, combined HCC-cholangiocarcinoma, cHCC-CCA, hepatitis B virus, HBV, Liver Imaging Reporting and Data System (LI-RADS)

## Abstract

The diagnostic performance of the Liver Imaging Reporting and Data System (LI-RADS) in differentiating hepatocellular carcinoma (HCC) from other hepatic malignancies has not been investigated in Chinese patients with chronic liver disease from hepatitis B virus (HBV) infection. The aim of this study was to evaluate the accuracy of the LI-RADS version 2018 in differentiating HCC, intrahepatic cholangiocarcinoma (ICCA), and combined HCC-cholangiocarcinoma (cHCC-CCA) in Chinese patients with HBV infection. Seventy consecutive HBV-infected patients with ICCA (n = 48) or cHCC-CCA (n = 22) who underwent contrast-enhanced magnetic resonance imaging (CE-MRI) between 2006 and 2017 were enrolled along with a comparison cohort of 70 patients with HCC and CE-MRI-matched for tumor size (10–19 mm, 20–30 mm, 31–50 mm, and >50 mm). Imaging feature frequencies for each tumor type were compared using Fisher’s exact test. The classification accuracy of LR-5 and LR-M was estimated for HCC versus non-HCC (ICCA and cHCC-CCA). The interobserver agreement was good for LI-RADS categories of HCC and moderate for non-HCC. After consensus read, 66 of 70 (94%) HCCs were categorized LR-5 (including tumor in vein [TIV] with LR-5), while 42 of 48 (88%) ICCAs and 13 of 22 (59%) cHCC-CCAs were categorized LR-M (including TIV with LR-M) (*p* < 0.001). Thus, assignment of LR-5 provided 94% sensitivity and 81% specificity for HCC. LR-M provided 79% sensitivity and 97% specificity for non-HCC (ICCA and cHCC-CCA); and the sensitivity and accuracy were lower in differentiating HCC from non-HCC (tumor size <20 mm). LI-RADS v2018 category 5 and M reliably differentiated HBV-related HCC from ICCA. However, a substantial proportion of cHCC-CCAs were categorized LR-5 rather than LR-M. While management is controversial for these combined tumors, accurate prospective differentiation is desired for optimal treatment.

## INTRODUCTION

Hepatocellular carcinoma (HCC) is the most common primary liver cancer and the second leading cause of cancer-related death in the world [1]. Hepatitis B and C viruses (HBV and HCV) are the important risk factors for HCC development and account for more than 80% of HCC cases worldwide [2]. Recently, HBV infection has also been implicated as a common risk factor for intrahepatic cholangiocarcinoma (ICCA) and combined HCC-cholangiocarcinoma (cHCC-CCA) development, particularly in HBV-endemic areas [3,4]. As we know, surgical resection and liver transplantation are effective and potentially curative options for HCC in cirrhotic patients, and nonsurgical oncologic interventions including various types of ablation, chemoembolization, and so on are available for HCC. However, ICCA is considered a contraindication for liver transplantation due to high recurrence rates, and surgical resection alone offers prolonged survival for patients with ICC, with little reported role of oncologic interventions. In addition, management is controversial for these combined tumors [5-7]. The treatment options and prognoses for patients with ICCA and cHCC-CCA differ from those with HCC and therefore, noninvasive differentiation of HCC from other primary hepatic malignancies is important. The overlap in imaging appearances of the different tumor types, especially in the setting of chronic liver disease and cirrhosis, may challenge accuracy of imaging diagnosis [8,9].

The liver imaging reporting and data system (LI-RADS) is a comprehensive algorithm which provides tools for standardizing the imaging diagnosis of patients at risk for HCC, and it is now completely concordant with the American Association for the Study of Liver Diseases (AASLD) guidance for the definite diagnosis and management of HCC [10,11]. The latest versions of LI-RADS (LI-RADS v2017 and 2018) [12], in addition to updated criteria for HCC diagnosis, also provide precise imaging criteria for assigning category LR-M (probable or definite malignancy, not specific for HCC). LR-M features are based predominately on the imaging characteristics of ICCAs but have also been reported in cHCC-CCAs [13]. The LR-M categorization intends to preserve the specificity of the LR-5 category for HCC without loss of sensitivity for the detection of malignancy [13]. Prior studies have demonstrated the diagnostic performance of LI-RADS version 2014 (LI-RADS v2014) in differentiating HCC from non-HCC malignancy in Western cohorts presenting with various chronic liver diseases, of which HCV was the most common [14,15]. However, chronic HBV accounts for more than two-thirds of HCCs in Asian countries. The overall survival rate is higher in HBV-associated HCC compared with HCV-associated HCC and this is likely due to better liver parenchymal reserve and less severe hepatic inflammation [16]. To the best of our knowledge, LI-RADS diagnostic accuracy studies for differentiating HCC from other hepatic malignancies have not been reproduced in a Chinese population with chronic liver disease from HBV infection. Therefore, the purpose of this study was to investigate the accuracy of LI-RADS v2018 to discriminate among cHCC-CCA, ICCA, and HCC in a Chinese population with chronic HBV infection.

## MATERIALS AND METHODS

### Study population

**FIGURE 1:**
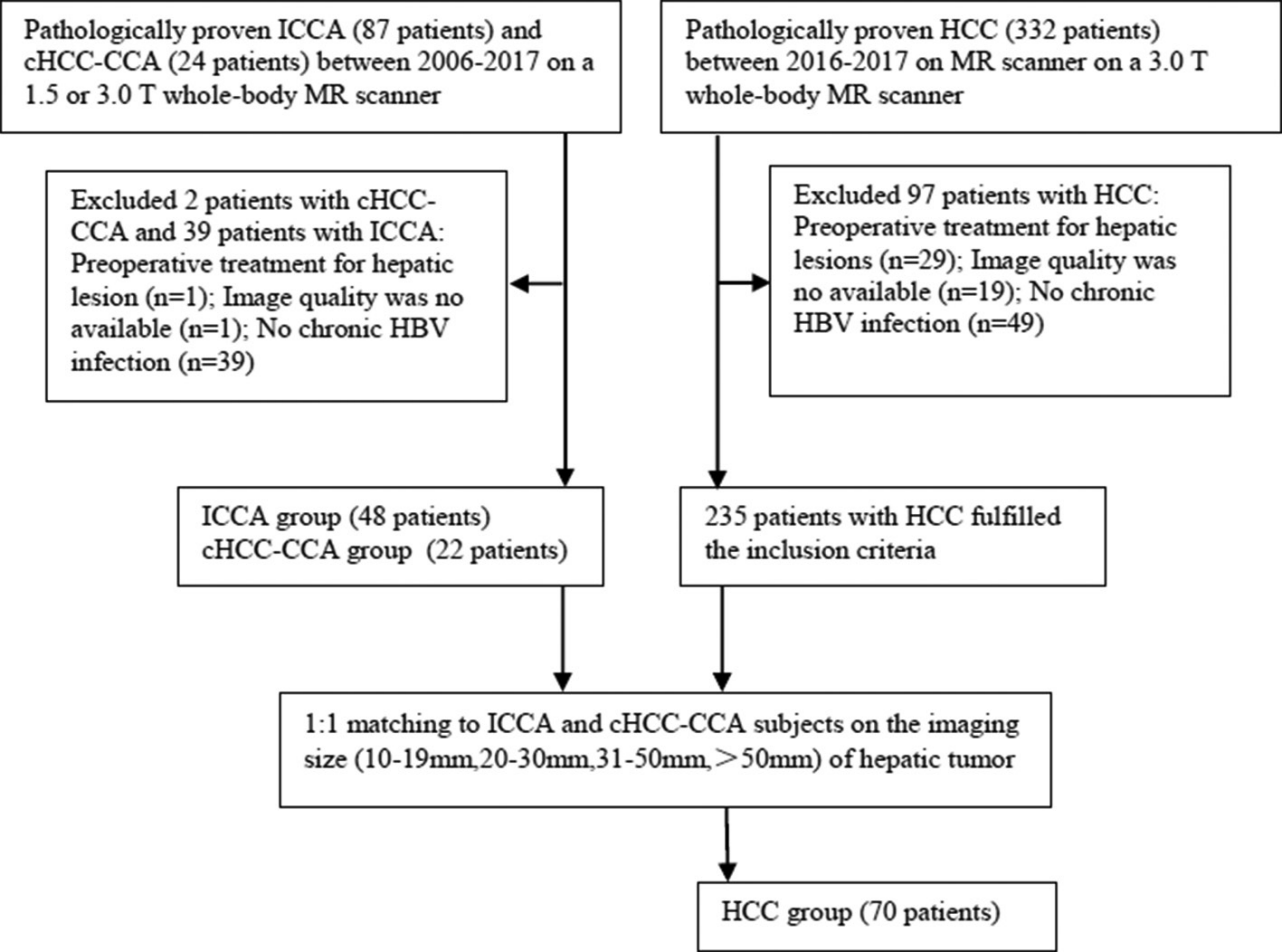
Flowchart showing the patient selection process. HCC: Hepatocellular carcinoma; ICCA: Intrahepatic cholangiocarcinoma; cHCC-CCA: Combined HCC-cholangiocarcinoma; HBV: Hepatitis B virus; MR: Magnetic resonance.

This retrospective study was approved by the institutional review board of our institution with waiver of written informed consent requirement. From November 2006 to December 2017, patients with either cHCC-CCA or ICCA who met the following criteria were enrolled: a) multiphase contrast-enhanced (CE)-magnetic resonance imaging (MRI) on 1.5 T or 3 T MR scanner performed within 30 days before tissue sampling; b) image quality was acceptable as defined below; c) chronic HBV infection; and d) no history of any previous therapy for liver malignancy. For the control group, during the latter 1 year of the study period (between 2016 and 2017), patients meeting the above categories of the inclusion criteria were enrolled by matching them one-to-one with the patients with non-HCCs (ICCA and cHCC-CCA) according to tumor imaging size (10–19 mm, 20–30 mm, 31–50 mm, and >50 mm). Patient clinical information and laboratory tests were obtained from retrospective review of the medical record. Figure 1 shows the flowchart of patient selection.

### MR image acquisition

Patients were scanned supine on a 3T whole-body MR scanner (Discovery MR750, GE Healthcare, Milwaukee, WI) and 1.5T whole-body MR scanner (GE Signa EXCITE HDxt, GE Healthcare, Waukesha, WI; Philips Achieva) with an eight-channel phased-array coil centered over the liver. Precontrast sequences included breath-hold coronal fast imaging employing steady-state acquisition (FIESTA), breath-hold coronal single-shot fast spin echo (SSFSE), respiratory-triggered axial T2-weighted fast spin echo (FSE), breath-hold two-dimensional dual-echo T1-weighted gradient-recalled-echo images at nominal opposed/in phase echo times for 1.5 T and 3 T, and respiratory-triggered axial diffusion-weighted spin-echo echo-planar imaging with 2 b values (b = 0 and 800 sec/mm^2^). Afterwards, breath-hold 3D T1W gradient-recalled-echo imaging (liver acquisition with volume acceleration) was performed before and at multiple time points dynamically after injection of gadobenate dimeglumine (Bracco), gadopentetate dimeglumine (Bayer), or gadoxetate disodium (Bayer). A dual arterial phase (AP) was initiated 15–20 seconds after the contrast media arrived at the distal thoracic aorta using bolus triggering, a portal venous phase (PVP) was acquired at 1 minute after contrast injection, and a delayed phase (DP)/transitional phase (TP) was acquired at 3 minutes. Twelve MRIs included delayed hepatobiliary phase imaging.

### Image analysis

All images were analyzed by two abdominal radiologists with 23 and 6 years of experience in liver MRI on a workstation of the picture archiving and communication system (PACS). The radiologists were blinded to the radiology report and histopathologic diagnosis, but they were aware that each patient had a diagnosis of HCC, ICCA, or cHCC-CCA. In cases of patients with multiple pathologically confirmed lesions, the largest tumor was evaluated for each patient. Before reviewing the MR images, both radiologists were given 3 months of hands-on instruction regarding the details of LI-RADS v2018 [12]. LI-RADS features that involve longitudinal assessment (threshold growth, subthreshold growth, size stability, and size reduction) were not applicable in this study as only one scan was evaluated per patient. In addition, due to the small number of exams performed with gadoxetate disodium, hepatobiliary phase LI-RADS features were not analyzed.

**FIGURE 2:**
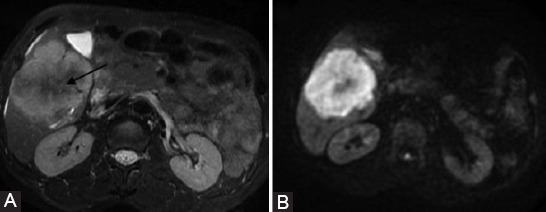
Targetoid appearance in T2WI-FS (A) and diffusion-weighted imaging [DWI] (B). A surgically confirmed combined hepatocellular carcinoma-cholangiocarcinoma (cHCC-CCA) in a 50-year-old male with hepatitis B virus infection. Concentric pattern in T2WI-FS and DWI characterized by mild-moderate hyperintensity in observation periphery with milder hypointensity in the center (arrow).

The LI-RADS imaging features were subclassified as a) major features (MF) of HCC (non-rim arterial phase hyperenhancement [APHE], nonperipheral washout appearance, and enhancing capsule appearance); b) ancillary features (AFs) that may favor malignancy in general (restricted diffusion, mild-moderate T2 hyperintensity, corona enhancement, iron sparing in solid mass, and fat sparing in solid mass); c) AFs that favor HCC in particular (nodule-in-nodule, mosaic architecture, blood products, and fat in mass); d) targetoid LR-M features (targetoid appearance on diffusion-weighted imaging [DWI], rim APHE, peripheral washout, and delayed central enhancement); and e) nontargetoid LR-M features (infiltrative appearance and necrosis or ischemia). In addition, three non-LI-RADS features were scored: liver surface retraction, biliary obstruction, targetoid appearance on T2WI (defined as concentric pattern in T2WI characterized by mild-moderate hyperintensity in observation periphery with relatively milder hyperintensity in the center, Figure 2). LI-RADS categories were assigned according to MFs and targetoid LR-M features. After initial independent review, consensus was reached on imaging features assessment and the LI-RADS categories (LR-4, LR-5, LR-M, and LR-TIV) in all observations.

### Histological analysis

All pathologic specimens were reviewed by a pathologist with 10 years of experience who specializes in pathologic diagnosis of hepatic diseases. The histopathologic assessment to differentiate HCC, ICCA, and cHCC-CCA was based on having hepatocellular, biliary, or both features, respectively. The diagnosis of cHCC-CCA was based on morphologic features and immunohistochemical findings (cytokeratins, *in situ* hybridization for albumin) of biphenotypic differentiation [17].

### Statistical analysis

Clinical characteristics, laboratory tests, and imaging features were compared using the Student’s t-test or Mann–Whitney U test for continuous variables and the Chi-square or Fisher exact test for categorical variables. The paired data of tumor size were compared using Wilcoxon signed ranks test. The diagnostic accuracy, sensitivity, and specificity of LR-5 and LR-M were estimated for HCC versus non-HCC malignancies (ICCA and cHCC-CCA). A test with *p* < 0.05 was considered statistically significant. Interobserver agreement was assessed by the Cohen κ statistic. Agreement was scored as follows [18]: poor (k, 0.00), slight (k = 0.00–0.20), fair (k = 0.21–0.40), moderate (k = 0.41–0.60), substantial (k = 0.61–0.80), and almost perfect (k = 0.81–0.99). All statistical analyses were performed using IBM SPSS Statistics for Windows, Version 22.0. (IBM Corp., Armonk, NY, USA).

## RESULTS

### Clinical characteristics

The final study population comprised 140 patients, 70 with non-HCC malignancies (22 cHCC-CCAs and 48 ICCAs) and 70 size-matched HCCs. The median interval between MRI and surgical resection or biopsy was mean 8 days (range 1–29 days). The demographic and clinical details are provided in Table 1. Alpha-fetoprotein (AFP) was significantly higher in HCC and cHCC-CCA than ICCA group. Carbohydrate antigen 19-9 (CA19-9) was significantly higher in ICCA and cHCC-CCA than HCC group. Cancer antigen 125 (CA125) was significantly higher in ICCA than HCC group.

**TABLE 1:**
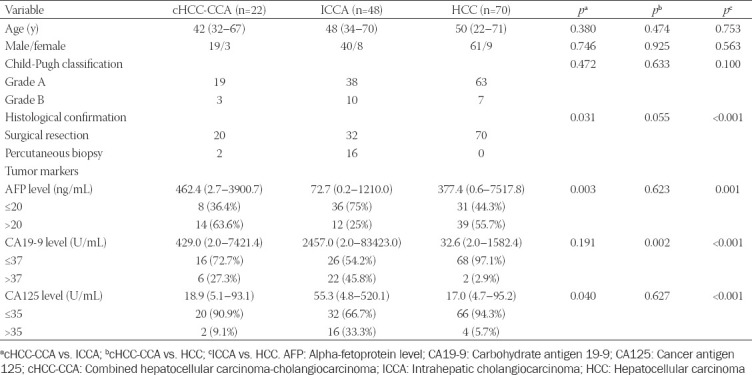
Clinical characteristics of the study patients among HBV-related cHCC-CCA, ICCA, and HCC

### Imaging features on LI-RADS v2018 and interobserver agreement

**FIGURE 3:**
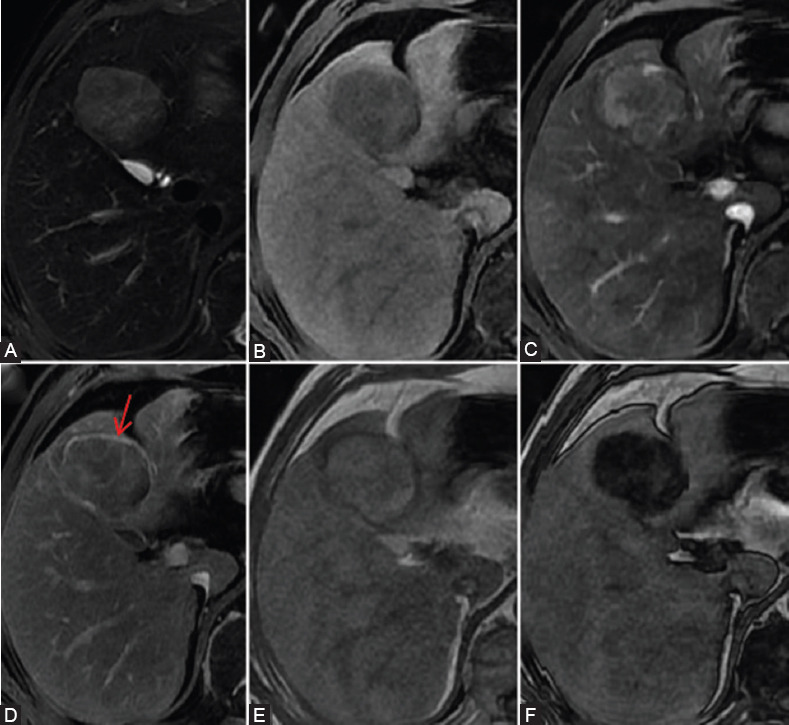
A surgically confirmed hepatocellular carcinoma (HCC) in a 53-year-old male with hepatitis B virus infection. (A) T2WI-FS image, (B) precontrast image, (C) late arterial, (D) delayed phase images, (E) dual-echo T1WI in-of-phase, and (F) out-of-phase sequences. Arterial phase hyperenhancement (not rim) during the late arterial phase and washout (not peripheral) during the delayed phase. (D) In delayed phase, the tumor showed enhancing capsule appearance (arrow). The tumor signal intensity decrease showed fat in mass on dual-echo T1WI out-of-phase sequence.

**FIGURE 4:**
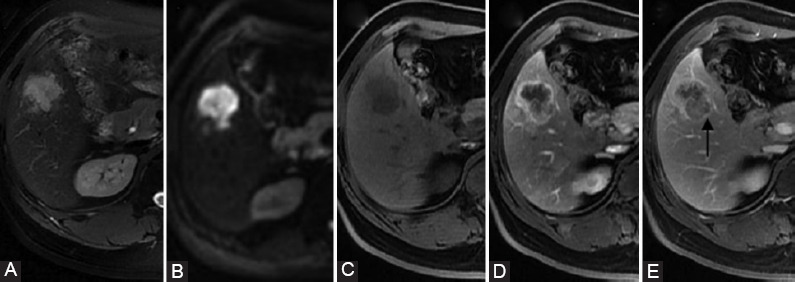
LR-M intrahepatic cholangiocarcinoma (ICCA). A surgically confirmed ICCA in a 46-year-old male with hepatitis B virus infection. (A) T2WI-FS image, (B) diffusion-weighted imaging (DWI) image (b = 1000 sec/mm), (C) precontrast image, (D) late arterial, and (E) delayed phase images. The tumor showed target appearance on DWI image. Rim arterial phase hyperenhancement during the arterial phase, progressive central enhancement during the delayed phase, and lobulated capsule appearance (arrow).

All consensus-assigned MFs of LI-RADS were significantly different in frequency among HBV-related cHCC-CCA, ICCA, and HCC (*p* < 0.05, Table 2). The MFs were significantly most frequent in HCC (Figure 3). Of the ancillary imaging features favoring HCC in particular, mosaic architecture was more frequent in HCC and cHCC-CCA than ICCA (*p* < 0.001 for both). Fat in mass was significantly more frequent in HCC than cHCC-CCA, HCC than ICCA, and cHCC-CCA than ICCA (*p* < 0.001 for all). By comparison, ICCA and cHCC-CCA showed significantly higher frequencies of LR-M features such as rim APHE, delayed central enhancement, and target appearance on DWI [all *p* < 0.005] (Figure 4). Peripheral “washout” was significantly more frequent in ICCA than HCC (*p* = 0.003). Necrosis or ischemia was significantly more frequent in ICCA than HCC and cHCC-CCA (all *p* < 0.05). In addition, ICCA and cHCC-CCA showed significantly higher frequencies of target appearance on T2WI, which is not currently one of ancillary features of LI-RADS v2018. ICCA showed significantly higher frequencies of liver surface retraction than HCC (*p* = 0.007). The interobserver agreement was good for LI-RADS categories for HCC; and moderate agreement was also achieved in the assignment of non-HCC malignancies (Table 3).

**TABLE 2:**
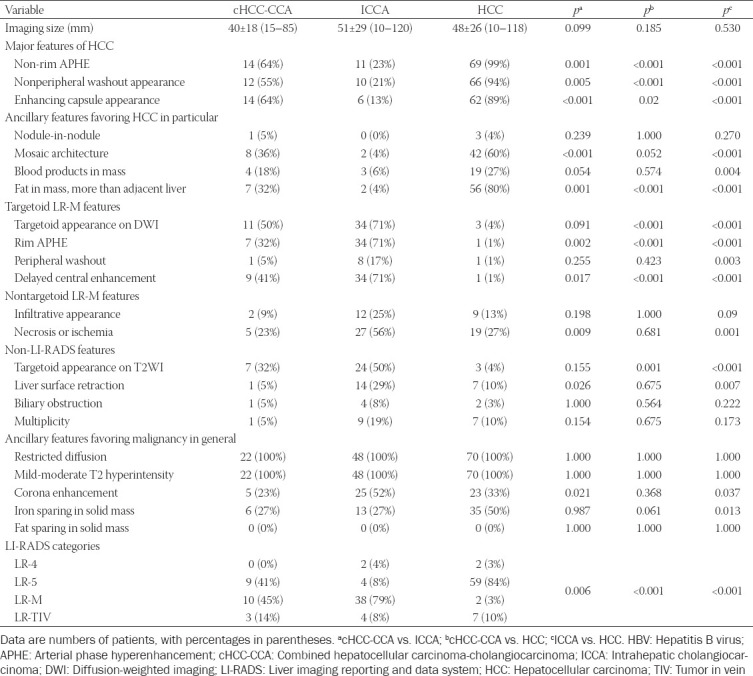
Imaging features after consensus read among HBV-related cHCC-CCA, ICCA, and HCC

**TABLE 3:**
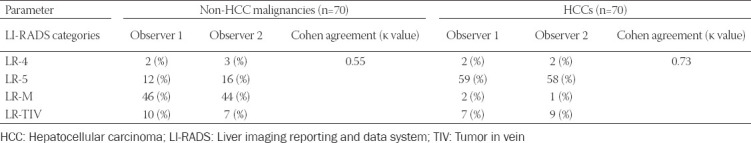
LI-RADS categories of the 140 observations and agreement between observers

### Diagnostic accuracy of LR-5 and LR-M for HCC versus non-HCC malignancies

Diagnosis performances of LR-5 and LR-M after consensus read for HCC or non-HCC are shown in Table 4. Assignment of LR-5 provided 94% sensitivity and 81% specificity for HCC. Four HCCs were categorized LR-4 (n = 2) or LR-M (n = 2). LR-M provided 79% sensitivity and 97% specificity for non-HCC (ICCA and cHCC-CCA). Six ICCAs were categorized LR-4 (n = 2) or LR-5 (n = 4), and nine cHCC-CCAs were categorized as LR-5. LI-RADS in combination with elevated tumor markers (AFP >100 ng/mL and CA 19-9 >100 U/mL, computed tomography [CT]/MRI LI-RADS® v2018 CORE, https://www.acr.org/Clinical-Resources/Reporting-and-Data-Systems/LI-RADS/CT-MRI-LI-RADS-v2018) could slightly improve diagnostic performance to differentiate HCC from non-HCC malignancies.

**TABLE 4:**
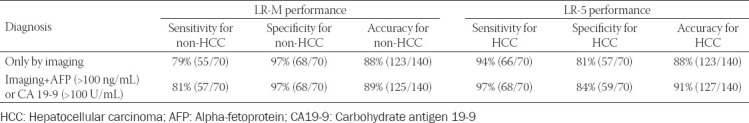
Diagnostic performance of LR-M and LR-5 after consensus read in the differential diagnosis between HCC and non-HCC

Table 5 shows that the sensitivity and accuracy were low in differentiating HCC from non-HCC in tumors with size <20 mm. The larger the tumors, the higher the sensitivity and accuracy were. LR-M demonstrated high specificity, sensitivity, and accuracy for tumors with a size >50 mm.

**TABLE 5:**
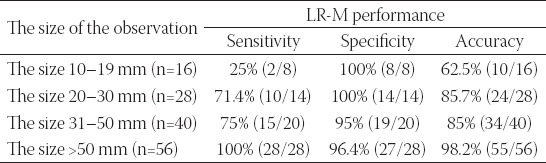
Diagnostic performance of LR-M according to the size of the observation

### Miscategorized HCC and non-HCC malignancies

**FIGURE 5:**
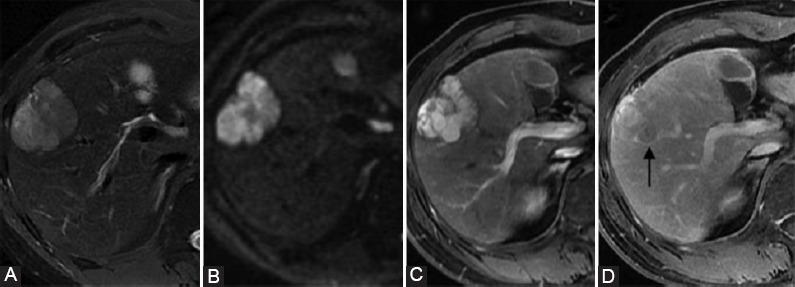
A surgically confirmed combined hepatocellular carcinoma-cholangiocarcinoma (cHCC-CCA) in a 43-year-old male with hepatitis B virus infection. (A) T2WI-FS image, (B) diffusion-weighted imaging (DWI) image (b = 1000 sec/mm), (C) late arterial, and (D) delayed phase images. The tumor showed high signal intensity on T2WI-FS and DWI images arterial phase hyperenhancement (not rim) during the late arterial phase and washout (not peripheral) during the delayed phase. (D) In delayed phase, the tumor showed lobulated capsule appearance (arrow).

**FIGURE 6:**
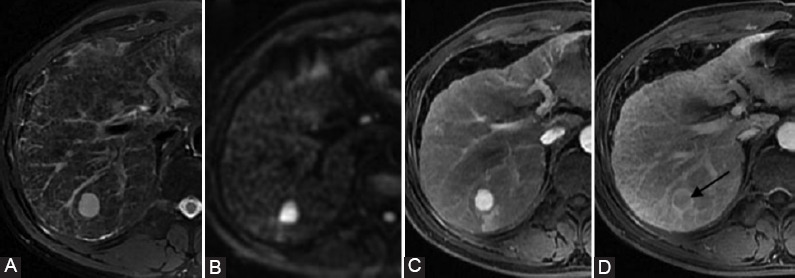
LR-5 intrahepatic cholangiocarcinoma (ICCA). A surgically confirmed ICCA in a 46-year-old male with HBV infection. (A) T2WI-FS image, (B) diffusion-weighted imaging (DWI) image (b = 1000 sec/mm), (C) late arterial, and (D) delayed phase images. The tumor showed high signal intensity on T2WI-FS and DWI images arterial phase hyperenhancement (not rim) during the late arterial phase and washout (not peripheral) during the delayed phase. (D) In delayed phase, the tumor showed enhancing capsule appearance (arrow).

Table 6 shows all cases that were miscategorized, along with features. All nine cHCC-CCAs showed at least two MFs of HCC and six cHCC-CCAs showed at least one AF favoring HCC (Figure 5). One had targetoid appearance on T2WI. All six ICCAs had non-rim APHE and four had nonperipheral “washout” (Figure 6). In addition, only one ICCA had fat in mass, but none were associated with any LR-M features.

**TABLE 6:**
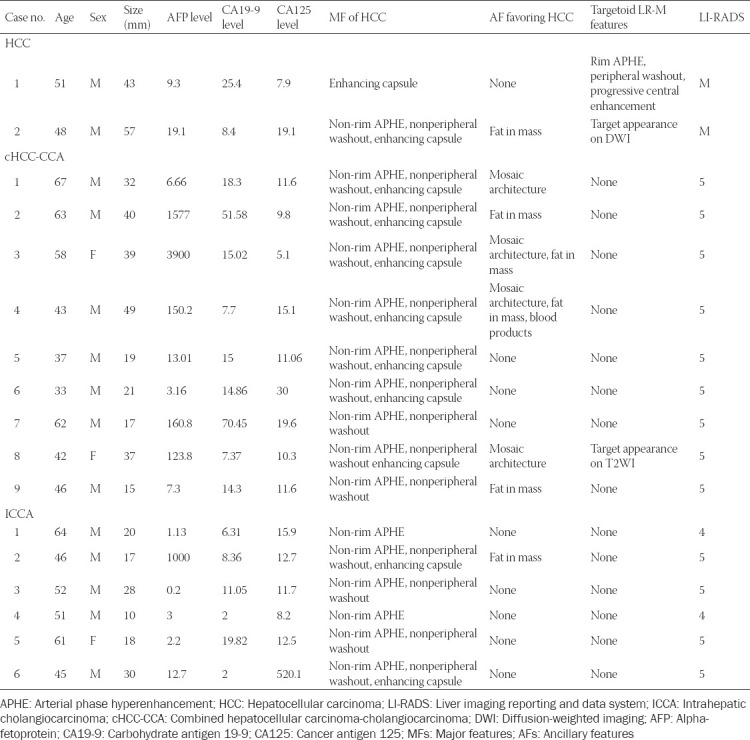
Characteristics of misclassified cases after consensus read based on LI-RADS

## DISCUSSION

The primary aim of our study was to evaluate the diagnostic accuracy of LR-5 and LR-M for differentiating HBV-related HCC from non-HCC malignancy. Using a large cohort of primary malignancies in an HBV population, we demonstrated that 94% HCCs were accurately categorized LR-5, while 88% ICCAs and 59% cHCC-CCAs were accurately categorized LR-M. Compared with the ideal of LI-RADS and results of other studies [19-21], we found a relatively modest specificity of LR-5 categorization for HCC (81%, compared to the provided range from the cited papers). The modest specificity of LR-5 for HCC in our study compared to the others likely reflects the enriched population of combined tumors in our cohort, which have been shown to demonstrate features of HC 4]. Ideally, no non-HCC malignancies would be categorized as LR-5. However, the literature consistently shows that the combined tumors and small tumors present a challenge to accurate diagnosis [9,14,15,22,23]. The diagnostic accuracy for identifying non-HCC malignancy was lower in smaller lesions (n = 16, tumor size 10–19 mm vs. n = 124, tumor size ≥20 mm). This has been shown in the literature [23,24] and is in part the reason for the imposed size thresholds in LI-RADS and other diagnostic algorithms.

LI-RADS v2018 was accurate for classifying most of HBV-related ICCA as non-HCC malignancy and proved high accuracy, specificity, and sensitivity, as has been previously reported [21]. Most HBV-related ICCA exhibited typical features including rim APHE, progressive central enhancement, peripheral washout, and target appearance on DWI, which were consistent with previous results [14,21,25]. However, six ICCAs (12.5%) were misclassified as LR-5 (n = 4) or LR-4 (n = 2), which showed smaller tumor size (1-3 cm) and non-rim APHE. Other studies have indicated that small ICCA in the setting of chronic viral hepatitis or cirrhosis may show non-rim APHE with the nonperipheral washout appearance, mimicking hypervascular HCC on contrast-enhanced CT and MRI [8,26,27]. Knowledge of the uncommon patterns of smaller ICCA enhancement is particularly important in transplant centers. We suggest that non-rim APHE lesions smaller than 3 cm in HBV-related liver should be carefully evaluated, and biopsy is necessary for the accurate diagnosis before the optimal treatment decision.

In our study, we used a novel imaging feature, T2WI targetoid appearance. T2WI targetoid appearance is not defined in LI-RADS but is similar to targetoid appearance on DWI and could be used at the discretion of the radiologist as a feature suggestive of a non-HCC malignancy, which is one route for making the LR-M categorization. In our cohort, targetoid appearance on T2WI had a statistical difference between non-HCC and HCC. Although the frequencies of target appearance on T2WI (31/70) were lower than targetoid appearance on DWI (45/70) in non-HCC malignancy (*p* = 0.018), this feature on T2WI has the potential supplementary value when some significant distortions appear in DWI images. Hence, we recommend it to be considered as a feature suggestive of non-HCC malignancy, though further research is needed to make it a distinct LR-M feature.

Similar to prior reports (6 of 14 [43%] and 4 of 11 [36.0%] biphenotypic primary liver carcinomas misdiagnosed as HCC) [28,29], in our cohort, 9 (41%) cHCC-CCAs met criteria for HCC according to MFs and had no AFs of a non-HCC malignancy. All seven exhibited APHE, washout appearance, and capsule appearance. Notably, capsule appearance, though significantly more common among HCCs, was identified for a substantial proportion (28.5%) of non-HCC malignancies (14 of 22 cHCC-CCAs and 6 of 48 ICCAs) in our cohort, consistent with the literature (13 of 42 [31.0%] non-HCC malignancies and 9 of 42 [21.4%] by two independent readers) [14]. We found that most capsules in non-HCC malignancy were presented as lobulated rims around the tumor.

While laboratory values are not currently incorporated into the diagnostic imaging algorithm, they may be helpful in challenging cases. As would be expected, AFP was significantly higher in cHCC-CCA than ICCA group, and CA19-9 was significantly higher in cHCC-CCA than HCC group. AFP and CA19-9 were simultaneously positive in the two miscategorized cHCC-CCA patients, which perhaps supports the possibility of cHCC-CCA. Ye et al. [30] similarly revealed that focal liver lesions mimicking HCC are observed in HBV-infected patients with elevated CA19-9, thus the possibility of cHCC-CCA should be considered. Other authors likewise have suggested that elevation in serum tumor markers that are discordant with the imaging findings may serve as a clue to the diagnosis of combined tumors [22].

Our retrospective study has several limitations. First, our study population was enriched to include only pathology-proven malignant lesions (either HCC or ICCA or cHCC-CCA), so they were not representative of a more general LI-RADS cohort which would be expected to have a spectrum of both benign and malignant observations. Second, our inclusion of 16 ICCAs from which tissue was obtained by percutaneous biopsy may have resulted in underrepresentation of cHCC-CCAs (e.g., if only the biliary portions of the mass were sampled). Of course, biopsy has been regarded as a confirmative tool that can determine the treatment plan in LI-RADS [10]. Third, threshold growth was excluded from image analysis in this study as only one scan was evaluated. Finally, given a smaller proportion of exams performed with hepatobiliary agents, the LI-RADS features on hepatobiliary phase were not evaluated.

## CONCLUSION

In conclusion, LI-RADS v2018 performed well in discriminating HCC from non-HCC malignancy in an Asian cohort of chronic HBV-infected patients. HBV-related ICCAs were misclassified in a minority of instances (small tumor size), whereas cHCC-CCA proved more challenging, i.e., a substantial proportion (41%) of cHCC-CCAs was miscategorized LR 5. In addition, T2WI targetoid appearance could be considered as a feature suggestive of non-HCC malignancy. For these atypical non-HCC malignancies, biopsy is recommended for the prospective accurate differentiation before the optimal treatment decision.
